# Interplays between Enterovirus A71 and the innate immune system

**DOI:** 10.1186/s12929-019-0596-8

**Published:** 2019-12-02

**Authors:** Kuan-Ru Chen, Pin Ling

**Affiliations:** 1 Department of Microbiology and Immunology, College of Medicine, National Cheng Kung Univeristy, Tainan, Taiwan; 2 Institute of Basic Medical Sciences, College of Medicine, National Cheng Kung Universiy, Tainan, Taiwan; 3 Department of Medical Laboratory Science and Biotechnology, College of Medicine, National Cheng Kung University, Tainan, Taiwan

**Keywords:** Enterovirus A71 (EV-A71), EV-A71 3C protease, EV-A71 2A protease, EV-A71 pathogenesis, TLRs, RLRs, NLRs

## Abstract

Enterovirus A71 (EV-A71) is a growing threat to public health, particularly in the Asia-Pacific region. EV-A71 infection is most prevalent in infants and children and causes a wide spectrum of clinical complications, including hand-foot-and-mouth disease (HFMD), pulmonary and neurological disorders. The pathogenesis of EV-A71 infection is poorly understood at present. It is likely that viral factors and host immunity, and their interplay, affect the pathogenesis and outcome of EV-A71 infection. The mammalian innate immune system forms the first layer of defense against viral infections and triggers activation of adaptive immunity leading to full protection. In this review, we discuss recent advances in our understanding of the interaction between EV-A71 and the innate immune system. We discuss the role of pattern-recognition receptors (PRRs), including Toll-like receptors (TLRs), RIG-I-like receptors (RLRs), and inflammasomes, in the detection of EV-A71 infection and induction of antiviral immunity. As a counteraction, EV-A71 viral proteins target multiple innate immune pathways to facilitate viral replication in host cells. These novel insights at the virus-host interphase may support the future development of vaccines and therapeutics against EV-A71 infection.

## Introduction

Enterovirus A71 (EV-A71) belongs to the Enterovirus genus in the Picornaviridae family and is a non-enveloped virus containing a positive single-stranded RNA (ssRNA) [1]. It was first identified in 1969 from children with the central nerve system-related complications [2]. EV-A71 infection causes outbreaks of hand-foot-and-mouth disease (HFMD) in infants and young children [3]. Severe cases are frequently associated with neurological complications like aseptic meningitis, acute flaccid paralysis, and encephalitis [3–5]. Clinical results indicated that deregulated inflammatory responses like cytokine storm might play a critical role in the EV-A71 pathogenesis [3]. At present, effective treatments and vaccines against EV-A71 are still warranted. Three formalin-inactivated EV-A71 vaccines for clinical treatment have been licensed in China [6–10]. In Taiwan, an inactivated EV-A71 vaccine has been developed and completed phase I and phase II studies [11, 12]. Additionally, human intravenous immunoglobulin (IVIG) is used for the treatment of EV-A71-associated brainstem encephalitis [13], but studies indicated that the antibody-dependent enhancement phenomenon is observed in EV71-infected patients [14–16].

EV-A71 has been shown to use scavenger receptor B2 (SCARB2) and P-selectin glycoprotein ligand-1 (PSGL-1) as entry receptors to establish infection in mammalian cells [17, 18]. Other molecules, including sialylated glycans [19], nucleolin [20], heparan sulfate glycosaminoglycan [21], and tryptophanyl-tRNA synthetase [22], are also shown to implicate in the infection of EV-A71 into mammalian cells. During EV-A71 infection, the positive sense ssRNA of EV-A71 encodes a large polyprotein, which in turn undergoes a series of cleavage processes to generate four structural viral proteins 1 to 4 (VP1-VP4) and seven nonstructural proteins (2A-2C and 3A-3D) [23]. EV-A71 2A protease cleaves between P1 and P2, whereas EV-A71 3C protease cleaves between P2 and P3 [23]. In addition to processing viral proteins, 2A and 3C are shown to target several host PRRs and innate immune regulators during infection. EV71 3D protein is transcribed as an RNA-dependent RNA polymerase (RDRP) to synthesize a complement negative strand in the cytoplasm [23].

The innate immune system elicits the first line of host defenses against pathogen infection meanwhile it links to the activation and programming of adaptive immune responses, leading to the full spectrum of immune protection. The host innate immune system detects invading pathogens by pattern-recognition receptors (PRRs) through recognizing conserved microbial components known as pathogen-associated molecular patterns (PAMPs) [24]. Several PRR families are existed in the mammalian innate immune system, including Toll-like receptors (TLRs), RIG-I-like receptors (RLRs), NOD-like receptors (NLRs), C-type lectin receptors [24–26], and cytosolic DNA sensors (DDX41, IFI16, and cGAS) [25, 27, 28]. Cytosolic innate immune regulators help to relay the PRR signals to the major downstream pathways, including NF-κB, MAPK, and/or IRF3/7, which in turn induce the production of inflammatory cytokines and/or type I interferons (IFNs) for mounting innate immune responses.

During viral infection, viral components, like viral proteins and viral nucleic acids, serve as PAMPs to be detected by PRRs to trigger antiviral innate immune responses. Several PRRs, such as endosomal TLRs (TLR3, TLR7/8, and TLR9), cytosolic RLRs (RIG-I and MDA5) and DNA sensors (DDX41, IFI16, and cGAS), detect viral nucleic acids to trigger downstream signaling pathways, resulting in the induction of type I IFNs and inflammatory cytokines [29, 30]. Type I IFNs are key for inducing effective antiviral immunity [31]. Type I IFNs serve two major functions at the early phase of viral infection. First, they activate hundreds of IFN-stimulated genes (ISGs) via the JAK-STAT pathways for mounting the antiviral state in virus-infected cells and neighboring cells [32, 33]. Second, they help dendritic cell maturation to enhance antigen presentation to T cells, then leading to viral antigen-specific adaptive immune responses [34]. Recent studies indicated that Type III IFNs can be produced earlier than type I IFNs by epithelial cells to controls viral infection at epithelial barriers [35]. It is also known that viruses develop sophisticated strategies to subvert antiviral innate immunity through targeting the PRR pathways and the JAK-STAT pathways [36, 37]. Further studies on decoding the complicated interactions between viruses and the mammalian innate immune system may cast insights toward the development of novel antiviral treatments. Here we review the current understanding of the mechanisms by which the mammalian innate immune system detects and responds to EV-A71 infection. In addition, we highlight the emerging roles of EV-A71 viral proteins in counteracting host innate immune pathways to promote viral propagation.

### A1, interplays between EV-A71 and the toll-like receptor pathways

**Fig. 1:**
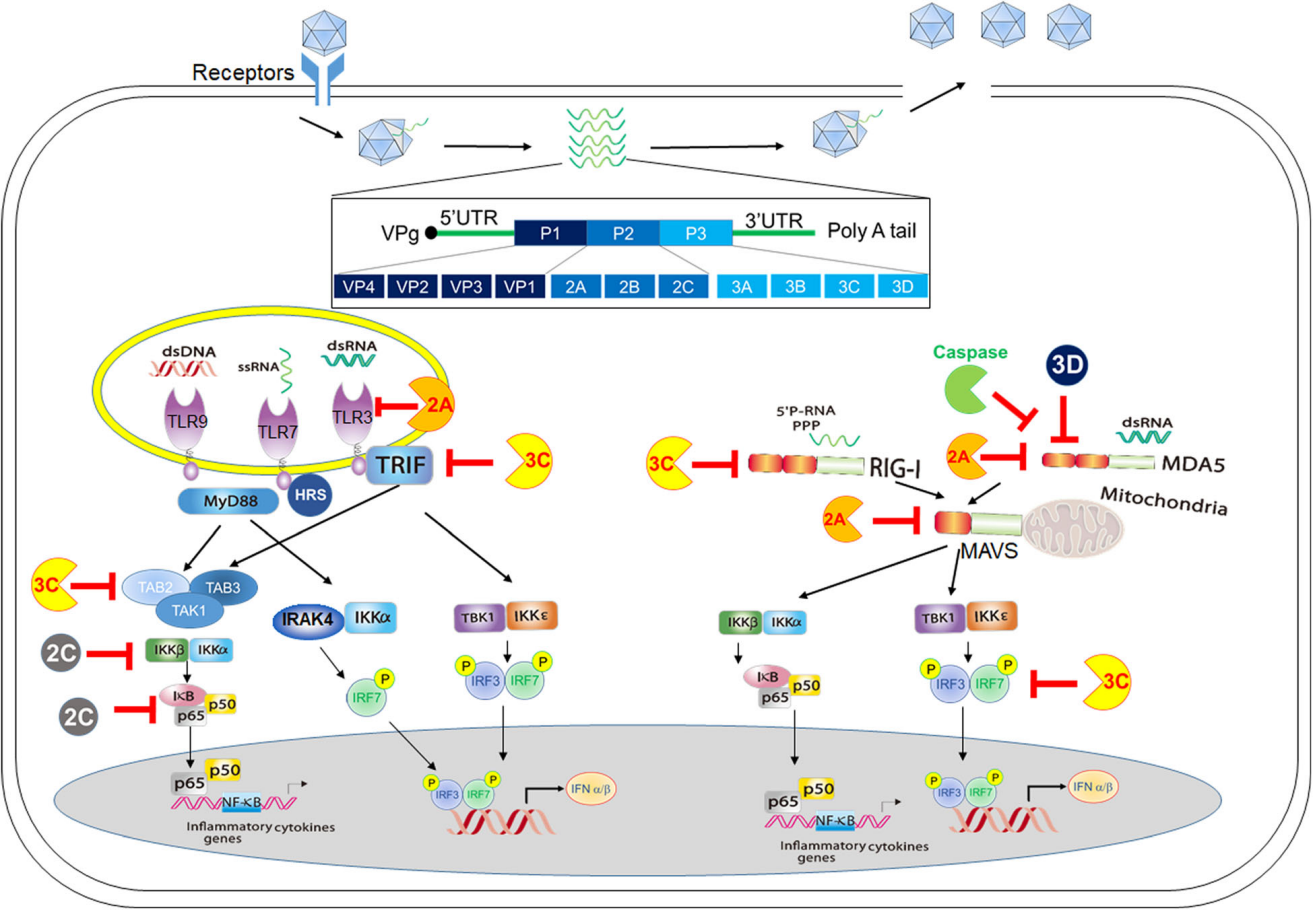
Interactions between EV-A71 and the TLR and RLR pathways. Upon EV-A71 infection, TLR3, TLR7, and MDA5 are implicated in detecting EV-A71 viral RNA in different cell types to trigger type I IFN-mediated antiviral immunity. TLR9 may detect endogenous host DNA from the lytic cycle of EV-A71-infected cells to trigger type I IFN-mediated antiviral immunity. To promote EV-A71 propagation in host cells, several EV-A71 viral factors, including 2A, 2C, 3C, and 3D, are shown to target the TLR3, RIG-I, and MDA5 pathways and downstream mediators to counteract antiviral innate immunity

The TLR family includes ten members in the human genome, and they can be categorized into two subgroups by their cellular distributions: cell surface TLRs and endosomal TLRs [31]. Cell surface TLRs are responsible for detecting microbial lipids, lipopeptides, and peptidoglycans from extracellular pathogens. Endosomal TLRs are located in the endolysosomal compartments to detect microbial nucleic acids from the endocytic, phagocytic, and autophagic pathways during pathogen infection. MyD88 is a key adaptor mediating downstream signaling in all TLR pathways except TLR3 [38, 39]. Trif is another adaptor critical for the endosomal TLR3 pathway and the endocytic branch of the TLR4 pathway [38, 39]. By far, endosomal TLRs have been well described to be involved in priming type I IFN-mediated antiviral responses against virus infection. We highlight the roles of TLRs in innate immune recognition of and defense against EV-A71 infection. The interplays between TLRs and EV-A71 infection are illustrated in Fig. 1 and Table 1.

**Table 1:** PRRs detect EV-A71 infection

PRRs	Cell lines and types	PAMPs and sensing mechanisms	References
TLR4	Human Mo-DCs, TLR4-transfected HEK293 cells	Viral particles	[40]
TLR3	Human Mo-DCs, Mouse BMMs, TLR3-transfected HEK293 cells	dsRNA	[41–43]
MDA5	Mouse MEFs, MDA5-transfected HeLa cells	dsRNA	[44, 45]
TLR7	THP-1, Mouse BMMs	ssRNA	[46]
NLRP3	Human PBMCs, Mouse BMDCs, THP-1, Mono Mac 6	1. EV-A71 2B protein induces the redistribution of NLRP3 2. EV-A71 3D protein interacts with the LRR domain of NLRP3	[47–49]
AIM2	SK-N-SH	Not determined	[50]
TLR9	Mouse pDCs, TLR9-transfected HEK293 cells	Host DNA from dying EV-A71-infected cells	[51]

*Human Mo-DCs* human monocyte-derived DCs, *HEK293* human embryonic kidney 293, *MEFs* mouse embryonic fibroblasts, *THP-1* human monocytic cell lines, *BMMs* bone marrow-derived macrophages, *PBMCs* peripheral blood mononuclear cells, *BMDCs* bone marrow-derived dendritic cells, *SK-N-SH* human neuroblastoma, *Mono Mac 6* human monocytic cell line, *pDCs* plasmacytoid DCs

A recent study revealed that TLR3 is a key sensor to detect viral dsRNA during EV-A71 infection, and subsequently to trigger downstream signaling to type I IFN induction and antiviral responses [41]. This TLR3-mediated detection of EV-A71 is established in TLR3-transfected HEK293 cells, primary macrophages and dendritic cells [41]. Of note, TLR3 was also shown to be a target of EV-A71 infection [41]. This notion will be discussed further in section A2 below. Another study also indicated that EV-A71 replication is increased in a human colon cancer cell line HT-29 after Trif is depleted by siRNA. Studies using TLR3-deficient mice have also revealed that TLR3 plays a critical role in defending against several enterovirus infections, such as Coxsackievirus A16, Coxsackievirus B3, Coxsackievirus B4, EV-A71, and Poliovirus [42, 52–54]. Notably, Zhu et al. found that invariant natural killer T (iNKT) cells are a key immune cell population induced in young mice after EV-A71 infection, leading to the protection of mice from EV71 infection [42]. Their findings further indicated that in vivo activation of iNKT cells after EV-A71 infection relies on TLR3 signaling in macrophages [42]. Furthermore, susceptibility to EV-A71 infection was shown to be age-dependent in mouse models [42, 55]. Of interest, genetic association studies from patients suggest that TLR3 gene polymorphisms are associated with the severity of EV-A71 infection in Chinese children [56, 57].

TLR7 is highly expressed in plasmacytoid DCs (pDCs), which produce a large amount of type I IFNs during virus infection. TLR7 detects ssRNA in endosomes and then activates the MyD88-dependent pathway through IKKα and IRF7 to IFN-α production [24, 58]. Notably, TLR7 has been shown to recognize the GU-rich and AU-rich ssRNA species from vesicular stomatitis virus (VSV), flaviviruses, Coxsackie B virus, and influenza A virus [52]. Recent studies have revealed the emerging roles of TLR7 in response to EV-A71 infection. Luo et al. demonstrated that EV-A71 infection induces the production of proinflammatory cytokines via the TLR7-NF-κB axis in several cell types, including human monocytic THP-1 cells, mouse bone marrow-derived macrophages (BMMs), and TLR7-expressed HEK293T cells [46]. Further, endosomal adaptor HRS has been shown to play a regulatory role in the assembly of TLR7 complex at endosomes during EV-A71 infection, leading to protection against EV-A71 infection [46]. Another study showed that the treatment of a TLR7 agonist GS-9620 significantly reduces EV-A71 replication in a mouse model [59]. EV-A71 and Coxsackievirus A16 replication are increased in human bronchial epithelial (16HBE) cells via induction of autophagy, which in turn mediates the degradation of endosomes and the TLR7 complex [60]. Of note, a recent study indicated that the allele C at TLR-7 rs3853839 locus has strongly correlated the severity of HFMD caused by EV71 infection [61]. Given the importance of TLR7 signaling in pDCs for type I IFN-mediated antiviral responses, further studies are warranted to investigate the importance of TLR7 in protecting against EV-A71 infection in vivo.

Similar to TLR7, endosomal TLR9 is also highly expressed in pDCs and detects microbial CpG DNA to trigger the MyD88-IKKα pathway to IFN-α production. In addition, TLR9 recognizes DAMPs such as tumor-derived mitochondrial DNA, IgG-chromatin complexes and HMGB1 [62]. A recent study using TLR9-deficient mice demonstrated that TLR9 deficiency leads to increased susceptibility to EV-A71 infection in mice [51]. The cytokine profiles of the brain from TLR9-deficient mice after EV-A71 infection exhibit decreased type-I IFN production but the increased production of several cytokines, including IFN-γ, IL-6, IL-1β, MIP-1α, MCP-1 and IP-10 [51]. It is plausible that the protective role of TLR9 in EV-A71 infection is due to TLR9-mediated recognition of endogenous host DNA from dying EV-A71-infected cells to induce type I IFN-mediated antiviral responses.

In addition to detecting lipopolysaccharide (LPS) from Gram-negative bacterial infection., TLR4 is also shown to detect several viral proteins [63], such as VSV-G [64], Ebola virus GP [65], influenza HA [66], respiratory syncytial virus fusion protein [67], and dengue virus NS1 protein [68, 69]. Recent work showed that ectopic expression of TLR4 or TLR4 plus MD2 in HEK293 cells enables the detection of EV-A71 virus-like particles to induce the production of IL-8 [40]. EV-A71 virus-like particles were further shown to induce TLR4-mediated expression of surface markers (like CD80, CD86, CD83, CD40, CD54, and HLA-DR) and production of cytokines (IL-12 p70, IL-12 p40, and IL-10) in human monocyte-derived DCs [40]. It will be interesting to further explore whether TLR4 is critical for defending against EV-A71 infection in vivo.

### A2, EV-A71 viral proteins target the toll-like receptor pathways

EV-A71, like other viruses, has utilized multiple ways to subvert the host antiviral responses to successfully establish infection. Having discussed the above findings that several TLRs are involved in regulating antiviral responses to EV71 infection, here we also discuss the actions of EV-A71 on counteracting the TLR pathways. First, EV-A71 infection results in the reduction of the TLR3 protein level in human neuroblastoma SK-N-SH cells and TLR3-transfected HEK293 cells [41]. EV-A71 2A protease is responsible for the cleavage of TLR3 [41]. Further studies are required to determine the mechanistic mechanism of 2A-mediated cleavage of TLR3 in a direct or an indirect manner. Another study showed that EV-A71 infection leads to the selective reduction of Trif adaptor in HeLa and RD cell lines [70]. Further, 3C protease binds and cleaves Trif adaptor to inhibit TLR3 signaling to type I IFN induction, and the Q312- S313 amino acids on Trif are critical for 3C-mediated cleavage [70]. In contrast, the degradation of Trif by EV-A71 3C protease is not occurred in a human colon cancer cell line HT-29 [43, 70]. These data suggest that 3C-mediated cleavage of Trif might be cell context-dependent. EV-A71 3C protease is also shown to target IRF7, a key transcriptional factor for type I IFN activation, at the Q189-S190 site [71].

NF-κB is a family of transcriptional factors linking PRRs and cytokine receptors (like IL-1 and TNF-α) to inflammatory responses. Several lines of evidence reveal the interplays between the NF-κB pathway and EV-A71 viral proteins. 3C protease is shown to block the NF-κB pathway to proinflammatory cytokine production by targeting the TAK1/TAB1/TAB2/TAB3 complex [72]. EV-A71 2C targets IKKβ and p65 to suppress NF-κB activation [73–75]. In addition to viral factors, EV-A71 infection also induces the expression of host microRNA miR-146a, which in turn downregulates the expression of TRAF6 and IRAK1 involved in TLR signaling to type I IFN induction [76]. Interestingly, depletion of miR-146a in mice by the genetic knockout or specific antagomiR approach restores the expression of IRAK1 and TRAF6, leading to increased IFNβ production, inhibition of EV-A71 replication and the improved survival rate [76]. The intervention of the TLR pathways by EV-A71 infection described above is illustrated in Fig. 1 and Table 2.

**Table 2:** EV-A71 viral proteins target PRRs and innate immune regulators

Targeted PRRs and innate immune regulators	Viral factor-mediated mechanisms	References
TLR3	2A downregulates TLR3	[41]
TRIF	3C cleaves TRIF at Q312- S313	[43, 70]
RIG-I	1. 3C targets RIG-I 2. 3C degrades RIG-I	[77] [78]
MDA5	1. 2A degrades MDA5 2. Host caspase cleaves MDA5 3. 3D interacts with the MDA5 CARD region	[78] [44] [79]
MAVS	2A cleaves MAVS at Gly209, Gly251, and Gly265	[43, 80]
NLRP3	1. 2A cleaves NLRP3 at Q225-G226 2. 3C cleaves NLRP3 G493-L494	[48]
GSMD	3C cleaves GSDMD at Q193-G194	[81]
TRAF6/IRAK1	Host miR-146a downregulates TRAF6/IRAK1	[76]
TAB2/TAK1/TAB1/TAB3	3C cleaves TAB2 at Q113-S114, TAK1 at Q360-S361, TAB1 at Q414-G415 and Q451-S452, and TAB3 at Q173-G174 and Q343-G344	[72]
IKKβ/p65	2C targets IKKβ and p65	[73, 74]
IRF7	3C cleaves IRF7 at Q189-S190	[71]
IRF9	3C cleaves IRF9	[82]
IFNAR1	2A downregulates IFNAR1	[83]
ZAP	3C cleaves ZAP at Q369 -G370	[84]

### B1, interplays between EV-A71 and the RIG-I-like receptor pathways

The RLR family consists of three members, including RIG-I, MDA5, and LGP2. Both RIG-I and MDA5 are shown to serve as cytosolic RNA sensors for detecting RNA virus infection while LGP2 function still remains controversial [85, 86]. MDA5 recognizes long dsRNA or viral RNA lacking 2′-*O*-methylation at their 5′ cap, whereas RIG-I recognizes short dsRNA or viral RNA species containing 5′ triphosphates or 5′ diphosphates [86–89]. Upon RNA ligand binding, RIG-I and MDA5 recruit a mitochondrial adaptor MAVS to activate TRAF3- and TRAF6-mediated downstream pathways for activation of IFN-β and inflammatory cytokines, respectively [86]. RIG-I and MDA5 play differential roles in detecting several RNA viruses [86, 90]. RIG-I is responsible for sensing RNA viruses like influenza viruses, VSV, and Japanese encephalitis virus. MDA5 is critical for the recognition of picornaviruses such as encephalomyocarditis virus (EMCV). Some viruses like dengue virus and West Nile virus are recognized by both RIG-I and MDA5 [85, 91]. We discuss recent findings with respect to the interactions between EV-A71 and the RLR pathways (Fig. 1 and Table 1).

A previous study demonstrated that transfection of EV-A71-derived RNA, but not EV-A71 infection, induces phosphorylation of an IFN-β transcriptional factor IRF3 in HeLa cells [44]. Further, MDA5 knockdown impairs IRF3 phosphorylation and the activation of the IFN-β mRNA in HeLa cells upon EV-A71-derived RNA transfection [44]. In addition, ectopic expression of MDA5 or RIG-I enhances the activation of the IFN-β mRNA and IRF3 phosphorylation upon EV-A71 infection [44]. Another study revealed that mouse embryonic fibroblasts deficient in MDA5 or MAVS are impaired in the activation of the IFN-β promoter upon EV-A71 viral RNA transfection [45]. However, a study using the reconstitution approach showed that HEK293 cells enable to recognize EV-A71 infection to activate type I IFNs only after ectopic expression of TLR3 but not MDA5 or RIG-I [41]. These findings suggest that MDA5 is able to detect EV-A71 viral RNA and might be involved in the detection of EV-A71 infection in a cell type-dependent way. It is possible that the 5′-end of viral RNAs of picornaviruses are covalently conjugated to VPg protein, and thus interfere RIG-I-mediated RNA sensing [92]. In addition, a report indicated that arrestin domain-containing 4 (ARRDC4), a regulator of G-protein-coupled receptors, interacts with MDA5 to facilitate MDA5 ubiquitination and activation to produce proinflammatory cytokines during EV-A71 infection [93]. A genetic association study noted that a polymorphism of MDA5 (rs1990760) is associated with the severity of EV71 infection in children [94]. The in vivo role of MDA5 in EV-A71 infection remains to be further explored.

### B2, EV-A71 viral proteins target the RIG-I-like receptor pathways

Several studies have shown the molecular mechanisms by which EV-A71 viral proteins target the RLR pathways during EV-A71 infection. A previous study revealed that EV-A71 viral protein 1 is co-localized with mitochondria and then induces mitochondrial abnormalities, and 2A protease cleaves MAVS at the Gly209, Gly251, and Gly265 residues to suppress type I IFN activation [80]. Degradation of MAVS after EV-A71 infection has been found in HeLa, RD and HT-29 cells [43, 80]. Feng et al. first showed that EV-A71 infection causes the cleavage of RIG-I, MDA5, and MAVS, and recombinant mengoviruses carrying EV-A71 2A also cleave these RLR molecules [78]. Also, Kuo et al. reported that EV-A71 infection induces the cleavage of endogenous MDA5 in HeLa cells and this MDA5 cleavage relies on the caspase activity from host cells [44]. Yet, more studies are needed to understand the underlying mechanism by which EV-A71 induces the cleavage of MDA5 in host cells by 2A protease or an indirect strategy. In addition to 2A protease, EV-A71 3C protease has also been shown to target the RIG-I pathway. Lei et al. reported that EV-A71 3C protease interacts with RIG-I to suppress Type I IFN activation during EV-A71 infection [77]. The infection of EV-A71 or mengoviruses carrying EV-A71 3C protease leads to the cleavage of RIG-I [78]. Interestingly, other enteroviruses, like poliovirus and Coxsackievirus B3 (CVB3), also employed similar strategies to target MDA5 and MAVS by their 2A proteases and to target RIG-I by their 3C proteases [78], suggesting that enteroviruses may use the common mechanisms to subvert the RLR pathways. Another study reported that EV-A71 3D polymerase interacts with MDA5 to disrupt the engagement of MDA5, leading to the downregulation of MDA5 signaling [79]. Together, the interplays between EV-A71 and the RLR pathways are concisely summarized in Fig. 1 and Table 2.

### C1, interplays between EV-A71 and Inflammasomes

**Fig. 2:**
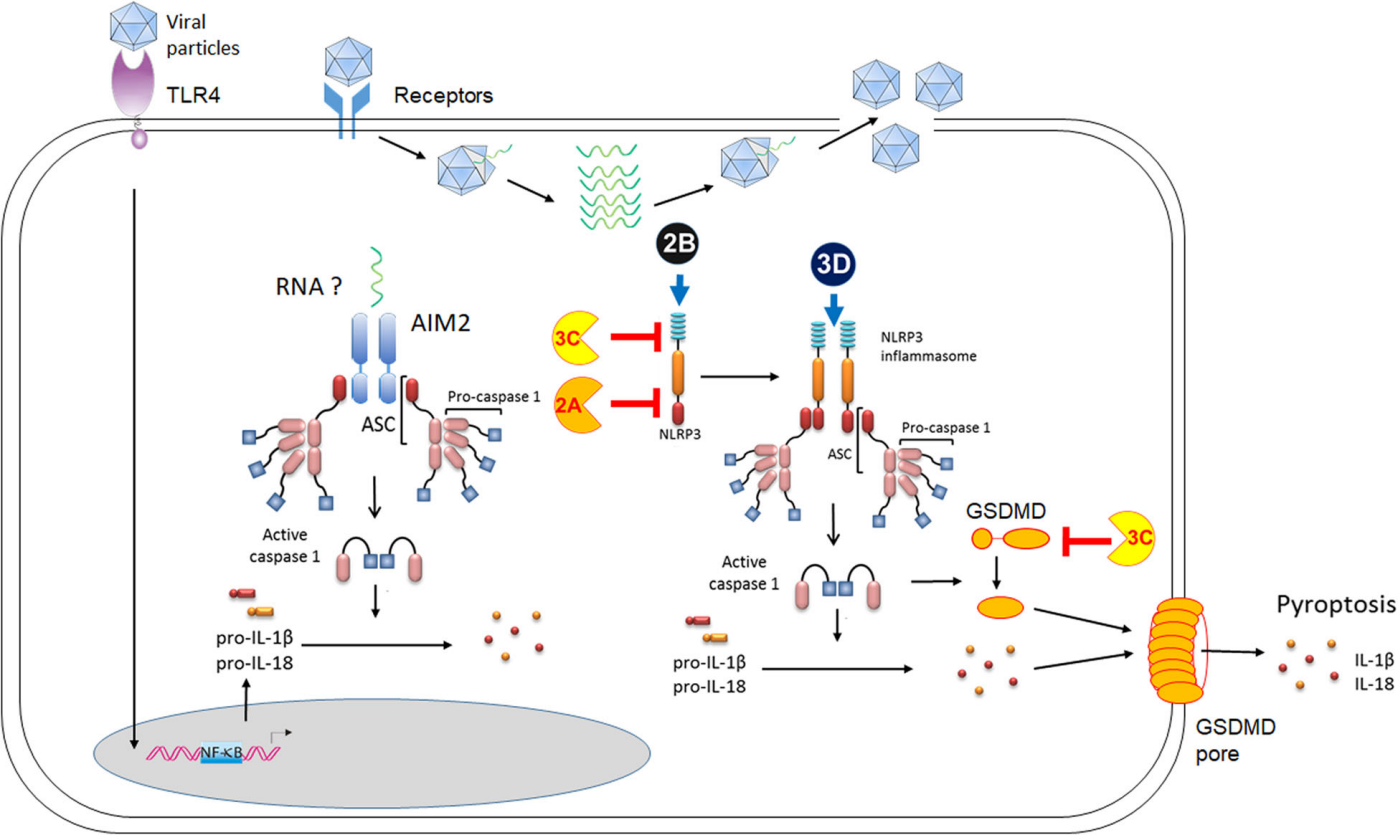
Interactions between EV-A71 and inflammasomes. Upon EV-A71 infection, EV-A71 2B and 3D interact NLRP3 to trigger NLRP3 inflammasome activation. AIM2 inflammasome is activated by the transfection of EV71 viral RNA. AIM2 plays a role in the restriction of EV-A71 replication. Meanwhile, EV-A71 viral factors 2A and 3C are shown to counteract NLRP3 inflammasome activation by targeting NLRP3 and GSDMD, respectively

Several NLRs function to form cytosolic inflammasomes to regulate innate immune responses to pathogen infection, tissue damage or metabolic stress [95, 96]. Among inflammasomes, NLRP3 inflammasome responds to a wide variety of PAMPs and DAMPs, and thus it has been under extensive investigations [96]. NLRP3 inflammasome activation requires two signals. The first signal is to activate the gene expression of pro-IL-1β, pro-IL-18, and NLRP3 via the PRR-NF-κB pathways. The second signal is to trigger the complex formation of the NLRP3 inflammasome by NLRP3 ligands, such as ATP, monosodium urate (MSU), pore-forming toxins, pathogen infection, and ultraviolet radiation [96]. Upon ligand stimulation, NLRP3 oligomerizes to recruit ASC and procaspase-1to form a large complex, then resulting in the activation of e caspase-1 via auto-cleavage [96]. Consequently, activated caspase-1 cleaves pro-IL-1β or pro-IL-18 to IL-1β or IL-18 for cytokine secretion [96]. Recent evidence indicated that gasdermin D (GSDMD) is another effector downstream of activated caspase-1, and the cleaved N-terminal portion of GSDMD can trigger pyroptosis and IL-1β secretion via its pore-forming activity [97–99]. Interactions between EV-A71 and inflammasomes have been demonstrated by recent work and briefly shown in Fig. 2 and Table 1.

Several studies showed that NLRP3 inflammasome activation occurs upon picornavirus infection, including EMCV, Rhinovirus, Poliovirus, CVB3, and EV-A71 [47, 100, 101]. Notably, mice deficient in inflammasome mediators, including NLRP3, ASC, Caspase-1 and IL-18, exhibited increased susceptibility to EV-A71 infection [48, 102]. Further evidence indicated that upon EV-A71 infection, NLRP3 inflammasome mediates the production of IL-1β from human monocytic cell lines (THP-1 and Mono Mac 6), human peripheral blood mononuclear cells (PBMCs), and mouse bone marrow-derived DCs [48]. One study noticed that EV-A71 3D protein, an RNA-dependent RNA polymerase (RdRp), interacts with the LRR domain of NLRP3 to facilitate the assembly of the inflammasome complex [49]. EV-A71 2B protein was found to induce the redistribution of NLRP3 to the perinuclear region and was co-localized with this redistributed NLRP3 [47]. It is likely that EV-A71 employs multiple mechanisms to engage with the NLRP3 inflammasome.

AIM2 is a cytosolic DNA sensor to detect cytosolic DNA from DNA viruses and other pathogens, and then recruits ASC and procaspase-1to form the inflammasome to trigger IL-1 maturation and pyroptosis [103]. A recent study indicated that AIM2 is highly expressed in the CNS tissues of human EV-A71 encephalomyelitis patients [50]. AIM2 gene expression is up-regulated by the transfection of EV-A71 RNA in SK-N-SH cells [50]. Silencing of AIM2 in SK-N-SH cells impaired the activation of IL-1 and increased viral replication upon EV-A71 infection [50]. The underlying mechanism of how EV-A71 activates the AIM2 inflammasome and the in vivo role of AIM2 during EV-A71 infection remains to be further explored.

### C2, EV-A71 viral proteins target the NLRP3 inflammasome

EV-A71 develops strategies to subvert inflammasome activation (Fig. 2 and Table 2). EV-A71 2A and 3C proteases were shown to cleave NLRP3 protein at the Q225-G226 pair or the G493-L494 pair respectively to suppress NLRP3 inflammasome activation [48]. Additionally, EV-A71 3C protease has also been shown to cleave GSDMD at the Q193-G194 pair to generate a shorter N-terminal GSDMD fragment (1–193 a.a.), leading to the inhibition of pyroptosis to facilitate EV-A71 replication [81].

### D1, interplays between EV-A71 and the IFN-ISG axis

Three types of IFNs exist in the mammalian immune system and play diverse roles in regulating innate and adaptive immunity. Among them, type I IFNs are major cytokines driving antiviral defense at the early stage of viral infection. The signals through type I IFN receptor (IFNAR) to induce expression of over 300 IFN-stimulated genes (ISGs), which play diverse roles in interfering viral replication in host cells at different steps [32, 33, 104]. Previous studies showed that treatment of type I IFNs on mice and mammalian cells confers antiviral immunity against EV71 infection [105, 106]. Studies using immunodeficient mouse models indicated that mice deficient in type I and/or type II IFN signaling become highly susceptible to EV-A71 infection [107–110]. Type III IFNs have been shown to play an important role in mucosal epithelial tissues to protect from viral attacks [111]. The natural route of EV-A71 infection is mainly through the gastrointestinal tract. Notably, a recent study indicated that EV-A71 infects human intestinal epithelium to produce type III IFNs (IFN-λ2/3), leading to the restriction of EV-A71 infection [112]. Also, EV71 was shown to be more sensitive to the treatment of IFN-λ3 than IFN-β [112]. It is conceivable that three types of IFNs may play their roles in distinct cell types to regulate immune responses to EV-A71 infection.

### D2, EV-A71 viral proteins target the IFN-ISG axis

**Fig. 3:**
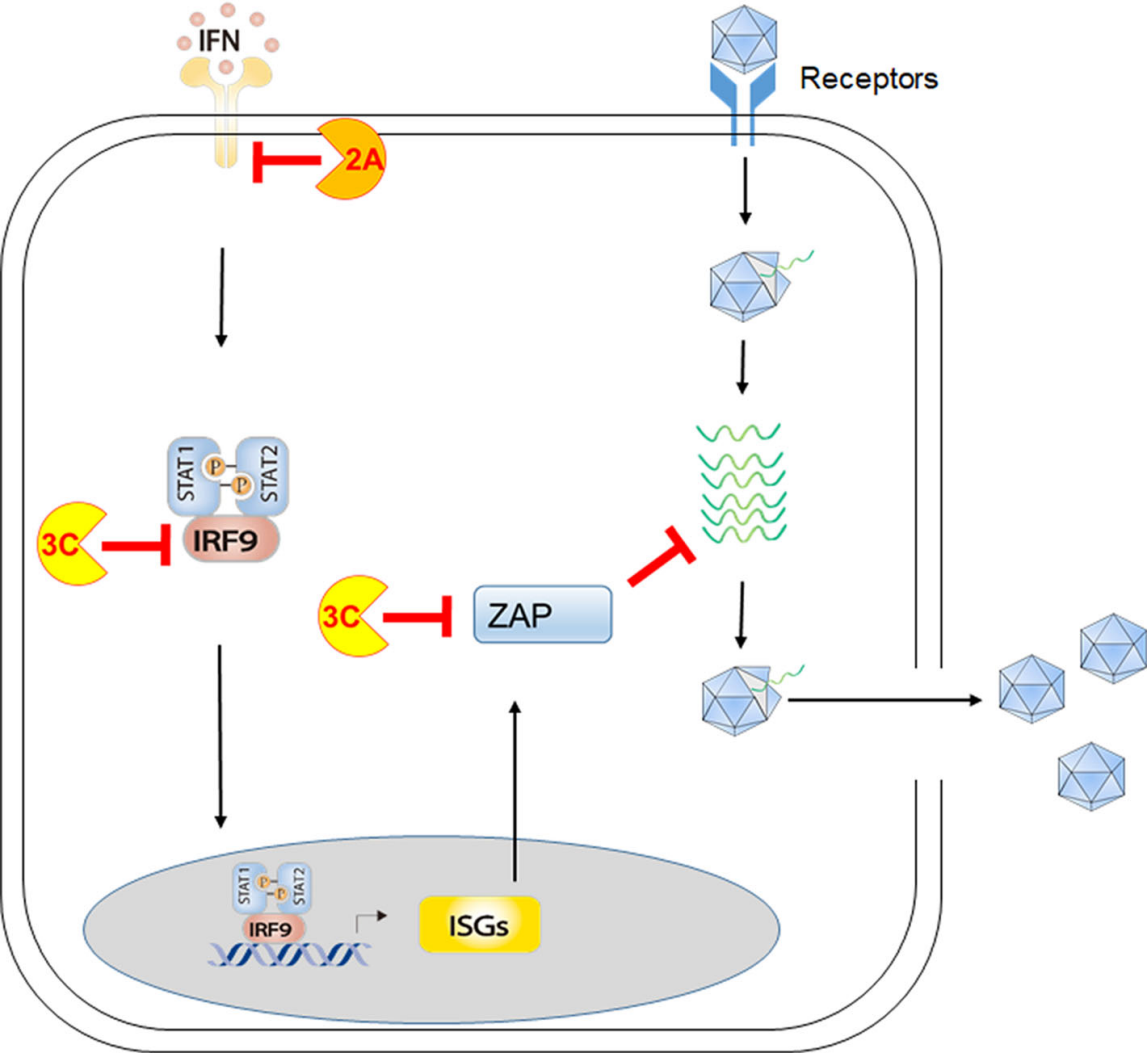
Interactions between EV-A71 and the type I IFN- signaling pathway. Upon cytokine engagement, type I IFN receptor (IFNAR) triggers the JAK-STAT pathway, leading to the activation of hundreds of IFN-stimulated genes (ISGs) to mount antiviral immunity. EV-A71 2A is shown to target IFNAR and IRF9 to curtail IFNR signaling to ISG induction. EV-A71 3C is shown to target one of ISGs called ZAP, which functions to inhibit viral replication.

Recent work also revealed the strategies of EV-A71 to antagonize the IFN-ISG axis. Lu et al. found that EV71 attenuates type I IFN signaling via its 2A protease to decrease the protein level of interferon receptor 1 (IFNAR1) [83]. EV-A71 3C protease was shown to cleave a transcriptional factor IRF9, which cooperates with STAT2 to mediate ISG expression [82]. A recent study showed that one of ISGs called Zinc-finger antiviral protein (ZAP) exhibits the ability to restrict EV-A71 replication, and EV-A71 3C protease cleaves ZAP at the Q369-G370 pair to diminish ZAP-mediated effect on EV-A71 replication [84]. Taken together, the interactions between EV-A71 and the IFN-ISG axis are illustrated in Fig. 3 and Table 2.

### Concluding remarks

Considerable progress has been made in understanding the complex interplays between EV-A71 and the innate immune system. Studies using cell lines or mouse models reveal that the mammalian innate immune system may employ multiple PRRs, including TLRs, RLRs, and inflammasomes, to sense the presence of EV-A71 to trigger innate immune responses in different cell types. EV-A71 has evolved multiple ways to subvert these PRR pathways and the IFN-ISG axis to promote viral propagation in host cells. Remarkably, the interactions between EV-A71 and some PRR pathways display a cell type-dependent effect. Further studies using primary cells, organoids, or conditional knockout mouse models may provide insightful knowledge toward understanding the importance of a given PRR pathway in EV-A71 infection. Of interest, insights gained from these studies could be potentially translated into clinic applications in the future. For instance, TLR9 ligand CpG has been used as an adjuvant for EV71 mucosal vaccine development [113]. NLRP3 ligand aluminum hydroxide has been included as an adjuvant for inactivated EV71 vaccine in phase III of a clinical trial [10]. In addition, antiviral drugs targeting EV-A71 factors, including protease inhibitors, 3D polymerase inhibitors, and 2C ATPase inhibitors, have been under development [114].

When we have gained certain knowledge about the interplays between EV-A71 and the innate immune system, however, several critical issues remain to be further explored. First, one of the major challenges in this field is lacking suitable mouse models to study EV-A71 infection through the oral-intestinal route. Future development of such models is critical to elucidate the unique operations of mucosal immunity during EV-A71 infection. Also, it will be more insightful to study the roles of tissue-specific innate immune cells in EV-A71 infection, such as microglia in the central nerve system, intestine-associated dendritic cells, macrophages, and innate lymphoid cells. Another critical issue is related to age-dependent immunity, which may affect the outcomes of EV71 infection [115, 116]. Further studies are needed to determine whether and how the subtle differences of early innate immunity in infants and adults may affect downstream host immune responses to EV-A71 infection. Lastly, it is imperative to translate knowledge gained from studying these critical issues toward the development of EV-A71 vaccines and antiviral therapies.

## Data Availability

Not applicable.

## Abbreviations

CAV16: Coxsackievirus A16
CVB: Coxsackievirus B
dsRNA: double-stranded RNA
EMCV: Encephalomeningitis virus
EV-A71: Enterovirus A71
HEK293: Human embryonic kidney 293
HFMD: Hand, foot and mouth disease
IFN: Interferon
ISGs: IFN-stimulated genes
PSGL-1: P-selectin glycoprotein ligand-1
RD: Rhabdomyosarcoma
RLRs: RIG-I-like receptors
SCARB: Scavenger receptor B2
ssRNA: single-stranded RNA
TLR: Toll-like receptor
iNKT: invariant natural killer T
ZAP: Zinc-finger antiviral protein
IVIG: Intravenous immunoglobulin
ADE: Antibody-dependent enhancement
PRRs: Pattern-recognition receptors
TBK-1: TANK-binding kinase 1
ISRE: Interferon-stimulated response element
pDCs: plasmacytoid DCs
VSV: Vesicular stomatitis virus
BMMs: Bone marrow-derived macrophages
HRS: Hepatocyte growth factor-regulated tyrosine kinase substrate
PBMCs: Peripheral blood mononuclear cells
DAMPs: Damage-associated molecular patterns
mtDNA: mitochondrial DNA
HT-29: Human intestinal epithelial cells
ARRDC4: Arrestin domain-containing 4
MSU: Monosodium urate
GSDMD: Gasdermin D
IFNAR: IFN receptor
